# 2,5-Dihexyl­thio­phene 1,1-dioxide

**DOI:** 10.1107/S1600536812046867

**Published:** 2012-11-24

**Authors:** Johannes Van Tonder, Mukut Gohain, Nagarajan Loganathan, Barend C. B. Bezuidenhoudt

**Affiliations:** a Department of Chemistry, University of the Free State, PO Box 339, Bloemfontein 9300, South Africa

## Abstract

In the title mol­ecule, C_16_H_28_O_2_S, the two *n*-hexyl groups are in all-*trans* conformations. Their C atoms are situated close to the plane of the thio­phene ring with a maximum deviation of 0.718 (6) Å for one of the terminal methyl groups. In the crystal, a short C—H⋯O contact is observed between thio­phene 1,1-dioxide groups.

## Related literature

For the preparation of the title compound, see: Barbarella *et al.* (1998 ▶). For a review on thio­phene-1,1-dioxide derivatives and their applications, see: Nakayama *et al.* (1999 ▶). For the biological activity of sulfone compounds, see: Naesens *et al.* (2006 ▶); Kim *et al.* (2008 ▶); Sagardoy *et al.* (2010 ▶). 
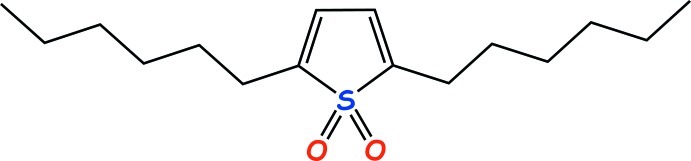


## Experimental

### 

#### Crystal data

C_16_H_28_O_2_S
*M* *_r_* = 284.44
Monoclinic, 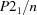

*a* = 5.8249 (11) Å
*b* = 11.248 (2) Å
*c* = 27.207 (6) Å
β = 91.770 (8)°
*V* = 1781.7 (6) Å^3^
*Z* = 4
Mo *K*α radiation
μ = 0.18 mm^−1^
*T* = 293 K
1.00 × 0.30 × 0.10 mm

#### Data collection

Bruker APEXII CCD diffractometer
Absorption correction: multi-scan (*SADABS*; Bruker 2008 ▶) *T*_min_ = 0.841, *T*_max_ = 0.982
18944 measured reflections
3149 independent reflections
2207 reflections with *I* > 2σ(*I*)
*R*_int_ = 0.072

#### Refinement

*R*[*F* ^2^ > 2σ(*F* ^2^)] = 0.062
*wR*(*F* ^2^) = 0.167
*S* = 1.07
3149 reflections
174 parameters
H-atom parameters constrained
Δρ_max_ = 0.26 e Å^−3^
Δρ_min_ = −0.20 e Å^−3^

### 

Data collection: *APEX2* (Bruker, 2008 ▶); cell refinement: *SAINT-Plus* (Bruker, 2008 ▶); data reduction: *SAINT-Plus*; program(s) used to solve structure: *SHELXTL* (Sheldrick, 2008 ▶) and *WinGX* (Farrugia 2012) ▶; program(s) used to refine structure: *SHELXTL*; molecular graphics: *DIAMOND* (Brandenburg & Putz, 2005 ▶); software used to prepare material for publication: *SHELXTL*.

## Supplementary Material

Crystal structure: contains datablock(s) I, global. DOI: 10.1107/S1600536812046867/gk2532sup1.cif

Structure factors: contains datablock(s) I. DOI: 10.1107/S1600536812046867/gk2532Isup2.hkl

Supplementary material file. DOI: 10.1107/S1600536812046867/gk2532Isup3.cml

Additional supplementary materials:  crystallographic information; 3D view; checkCIF report

## Figures and Tables

**Table 1:** Hydrogen-bond geometry (Å, °)

*D*—H⋯*A*	*D*—H	H⋯*A*	*D*⋯*A*	*D*—H⋯*A*
C3—H3⋯O2^i^	0.93	2.54	3.186 (3)	126

Symmetry code: (i) 
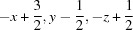
.

**Fig. 1.**
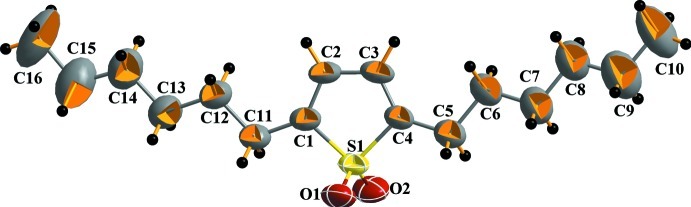
Molecular structure of the title compound with displacement ellipsoids shown at the 50% probability level.

## supplementary crystallographic information

### Comment

Tetrahydrothiophene 1,1-dioxide otherwise known as sulfolane, is an important industrial solvent used in the purification of natural gas, particularly for the extraction of aromatic hydrocarbon from the other hydrocarbon mixtures. Thiophene 1,1-dioxide is an important intermediate in the synthesis of various class of organic compounds. This class of compound is also known for their bioapplications (Nakayama *et al.*, 1999; Naesens *et al.*, 2006; Kim *et al.*, 2008; Sagardoy *et al.*, 2010). For example, some of them have been found to be effective inhibitors of hepatitis C virus polymerase (Kim *et al.*, 2008).

The title compound, 2,5-dihexyl-thiophene-1,1-dioxide, was synthesized by the procedure of Barbarella *et al.* (1998).

### Experimental

A mixture of 2,5-dihexylthiophene (0.500 g; 2.0 mmol) and NaHCO_3_ (0.667 g; 7.9 mmol; 4.0 eq) was taken in dichloromethane(30 ml) at 0 °C and allowed to stir vigorously for a few minutes. To which a freshly recrystallized (from dichloromethane) solid of *m*-chloroperbenzoic acid (1.411 g; 8.2 mmol; 4.1 eq.) was added in portion over 60 min. After 16hrs of stirring, the precipitate was removed by filtration and successively washed with dichloromethane (2 *x* 5 ml). The collective filtrate was then evaporated to dryness. Crystals of title compound were obtained as white needles from n-pentane. Yield: 0.304 g; 53.9%. ^1^H NMR (600 MHz, CDCl3) δ 6.28 – 6.24 (2*H*, m, H-3,4), 2.46 (4*H*, t, J = 7.7 Hz, H-1'), 1.68 – 1.61 (4*H*, m, H-2'), 1.41 – 1.34 (4*H*, m, H-3'), 1.33 – 1.26 (8*H*, m, H-4',5'), 0.91 – 0.86 (6*H*, m, H-6'). 13 C NMR (151 MHz, CDCl3) δ 144.08 (C-2,5), 121.80 (C-3,4), 31.51 (C-4',5'), 28.91 (C-3'), 26.67 (C-2'), 24.41 (C-1'), 22.63 (C-4',5'), 14.16 (C-6'). EI—MS m/*z*: 284.10 (35.47%; *M*+), 165.10 (78.57), 95.10 (100.00), 81.05 (83.06).

### Refinement

All H atoms were positioned geometrically with C—H distances in the range 0.93 - 0.97 Å. and allowed to ride on their parent atoms, with *U*_iso_(H) =1.2Ueq(C) except methyl group where *U*_iso_(H) = 1.5Ueq(C).

### Crystal data

**Table:**

C_16_H_28_O_2_S	*F*(000) = 624
*M**_r_* = 284.44	*D*_x_ = 1.060 Mg m^−^^3^
Monoclinic, *P*2_1_/*n*	Mo *K*α radiation, λ = 0.71073 Å
Hall symbol: -P 2yn	Cell parameters from 4797 reflections
*a* = 5.8249 (11) Å	θ = 2.4–23.1°
*b* = 11.248 (2) Å	µ = 0.18 mm^−^^1^
*c* = 27.207 (6) Å	*T* = 293 K
β = 91.770 (8)°	Needle, colourless
*V* = 1781.7 (6) Å^3^	1.00 × 0.30 × 0.10 mm
*Z* = 4	

### Data collection

**Table:**

Bruker APEXII CCD diffractometer	3149 independent reflections
Radiation source: sealed tube	2207 reflections with *I* > 2σ(*I*)
Graphite monochromator	*R*_int_ = 0.072
φ and ω scans	θ_max_ = 25.1°, θ_min_ = 2.4°
Absorption correction: multi-scan (*SADABS*; Bruker 2008)	*h* = −6→5
*T*_min_ = 0.841, *T*_max_ = 0.982	*k* = −13→13
18944 measured reflections	*l* = −32→32

### Refinement

**Table:**

Refinement on *F*^2^	Primary atom site location: structure-invariant direct methods
Least-squares matrix: full	Secondary atom site location: difference Fourier map
*R*[*F*^2^ > 2σ(*F*^2^)] = 0.062	Hydrogen site location: inferred from neighbouring sites
*wR*(*F*^2^) = 0.167	H-atom parameters constrained
*S* = 1.07	*w* = 1/[σ^2^(*F*_o_^2^) + (0.0703*P*)^2^ + 0.6152*P*] where *P* = (*F*_o_^2^ + 2*F*_c_^2^)/3
3149 reflections	(Δ/σ)_max_ < 0.001
174 parameters	Δρ_max_ = 0.26 e Å^−^^3^
0 restraints	Δρ_min_ = −0.20 e Å^−^^3^

### Special details

**Table:**

Experimental. Crystal was mounted and automatically centered on a Bruker *SMART* X2S bench top crystallographic system. Data were collected at 20°C with 60 s/frame exposure time (total of 1260, width 0.5°) covering up to θ = 25.11° and 99.9% completeness accomplished.
Geometry. All e.s.d.'s (except the e.s.d. in the dihedral angle between two l.s. planes) are estimated using the full covariance matrix. The cell e.s.d.'s are taken into account individually in the estimation of e.s.d.'s in distances, angles and torsion angles; correlations between e.s.d.'s in cell parameters are only used when they are defined by crystal symmetry. An approximate (isotropic) treatment of cell e.s.d.'s is used for estimating e.s.d.'s involving l.s. planes.
Refinement. Refinement of *F*^2^ against ALL reflections. The weighted *R*-factor *wR* and goodness of fit *S* are based on *F*^2^, conventional *R*-factors *R* are based on *F*, with *F* set to zero for negative *F*^2^. The threshold expression of *F*^2^ > σ(*F*^2^) is used only for calculating *R*-factors(gt) *etc*. and is not relevant to the choice of reflections for refinement. *R*-factors based on *F*^2^ are statistically about twice as large as those based on *F*, and *R*- factors based on ALL data will be even larger.

### Fractional atomic coordinates and isotropic or equivalent isotropic displacement parameters (Å^2^)

**Table:**

	*x*	*y*	*z*	*U*_iso_*/*U*_eq_	
S1	0.46906 (11)	1.12674 (5)	0.21332 (3)	0.0601 (3)	
O1	0.3088 (3)	1.17815 (16)	0.17855 (8)	0.0747 (6)	
O2	0.6195 (3)	1.20741 (15)	0.23951 (8)	0.0810 (7)	
C1	0.3264 (5)	1.0326 (2)	0.25486 (11)	0.0626 (7)	
C2	0.4059 (5)	0.9226 (2)	0.24780 (11)	0.0694 (8)	
H2	0.3564	0.8578	0.2659	0.083*	
C3	0.5755 (5)	0.9109 (2)	0.20979 (12)	0.0723 (9)	
H3	0.6418	0.8382	0.2022	0.087*	
C4	0.6296 (5)	1.0103 (2)	0.18666 (11)	0.0607 (7)	
C5	0.7877 (5)	1.0372 (2)	0.14685 (13)	0.0754 (9)	
H5A	0.8910	1.1002	0.1577	0.090*	
H5B	0.6990	1.0664	0.1186	0.090*	
C6	0.9293 (6)	0.9312 (3)	0.13111 (14)	0.0841 (9)	
H6A	1.0245	0.9051	0.1589	0.101*	
H6B	0.8260	0.8666	0.1222	0.101*	
C7	1.0796 (6)	0.9554 (3)	0.08902 (15)	0.0989 (11)	
H7A	1.1824	1.0204	0.0977	0.119*	
H7B	0.9846	0.9806	0.0610	0.119*	
C8	1.2227 (7)	0.8481 (4)	0.07397 (17)	0.1131 (13)	
H8A	1.3208	0.8246	0.1017	0.136*	
H8B	1.1196	0.7824	0.0665	0.136*	
C9	1.3646 (10)	0.8683 (5)	0.0321 (2)	0.157 (2)	
H9A	1.4668	0.9344	0.0396	0.189*	
H9B	1.2660	0.8918	0.0044	0.189*	
C10	1.5056 (10)	0.7652 (6)	0.0169 (2)	0.174 (2)	
H10A	1.6233	0.7500	0.0416	0.261*	
H10B	1.5751	0.7827	−0.0138	0.261*	
H10C	1.4093	0.6963	0.0131	0.261*	
C11	0.1562 (5)	1.0832 (2)	0.28819 (12)	0.0699 (8)	
H11A	0.0457	1.1296	0.2689	0.084*	
H11B	0.2345	1.1368	0.3110	0.084*	
C12	0.0284 (6)	0.9910 (3)	0.31714 (13)	0.0772 (9)	
H12A	−0.0550	0.9395	0.2942	0.093*	
H12B	0.1395	0.9423	0.3352	0.093*	
C13	−0.1368 (6)	1.0412 (3)	0.35249 (13)	0.0923 (10)	
H13A	−0.2437	1.0931	0.3348	0.111*	
H13B	−0.0528	1.0889	0.3767	0.111*	
C14	−0.2728 (7)	0.9448 (4)	0.37919 (16)	0.1160 (14)	
H14A	−0.3549	0.8971	0.3547	0.139*	
H14B	−0.1644	0.8929	0.3964	0.139*	
C15	−0.4357 (10)	0.9875 (5)	0.4140 (2)	0.155 (2)	
H15A	−0.5433	1.0410	0.3974	0.186*	
H15B	−0.3547	1.0321	0.4396	0.186*	
C16	−0.5686 (10)	0.8855 (7)	0.4373 (2)	0.192 (3)	
H16A	−0.6504	0.8417	0.4121	0.288*	
H16B	−0.6758	0.9174	0.4600	0.288*	
H16C	−0.4630	0.8336	0.4545	0.288*	

### Atomic displacement parameters (Å^2^)

**Table:**

	*U*^11^	*U*^22^	*U*^33^	*U*^12^	*U*^13^	*U*^23^
S1	0.0592 (4)	0.0319 (3)	0.0888 (5)	−0.0010 (3)	−0.0051 (4)	0.0004 (3)
O1	0.0735 (12)	0.0499 (10)	0.0999 (15)	0.0105 (9)	−0.0096 (11)	0.0143 (10)
O2	0.0808 (13)	0.0438 (10)	0.1173 (17)	−0.0150 (9)	−0.0117 (12)	−0.0124 (10)
C1	0.0659 (17)	0.0403 (13)	0.0810 (19)	−0.0032 (12)	−0.0056 (15)	0.0016 (12)
C2	0.081 (2)	0.0363 (13)	0.091 (2)	−0.0026 (13)	0.0022 (17)	0.0073 (13)
C3	0.0754 (19)	0.0374 (13)	0.103 (2)	0.0078 (13)	−0.0057 (18)	−0.0004 (14)
C4	0.0541 (15)	0.0411 (13)	0.0864 (19)	0.0029 (12)	−0.0040 (15)	−0.0038 (13)
C5	0.0680 (19)	0.0589 (16)	0.099 (2)	0.0019 (15)	−0.0018 (18)	−0.0043 (16)
C6	0.073 (2)	0.075 (2)	0.104 (2)	0.0089 (17)	0.0018 (19)	−0.0114 (19)
C7	0.088 (2)	0.100 (3)	0.109 (3)	0.008 (2)	0.014 (2)	−0.013 (2)
C8	0.103 (3)	0.119 (3)	0.118 (3)	0.013 (3)	0.022 (3)	−0.011 (3)
C9	0.147 (4)	0.176 (6)	0.150 (5)	0.038 (4)	0.033 (4)	−0.011 (4)
C10	0.158 (5)	0.202 (6)	0.164 (5)	0.069 (4)	0.033 (4)	−0.040 (5)
C11	0.0752 (19)	0.0464 (14)	0.088 (2)	−0.0014 (14)	0.0038 (16)	−0.0020 (14)
C12	0.081 (2)	0.0609 (17)	0.090 (2)	−0.0078 (15)	0.0033 (18)	0.0062 (16)
C13	0.099 (3)	0.086 (2)	0.093 (2)	−0.007 (2)	0.013 (2)	0.009 (2)
C14	0.108 (3)	0.135 (4)	0.106 (3)	−0.004 (3)	0.019 (3)	0.021 (3)
C15	0.147 (4)	0.189 (6)	0.130 (4)	−0.039 (4)	0.022 (4)	0.009 (4)
C16	0.152 (5)	0.277 (8)	0.149 (5)	−0.087 (5)	0.016 (4)	0.075 (5)

### Geometric parameters (Å, º)

**Table:**

S1—O1	1.4306 (19)	C9—H9A	0.9700
S1—O2	1.4353 (19)	C9—H9B	0.9700
S1—C1	1.774 (3)	C10—H10A	0.9600
S1—C4	1.777 (3)	C10—H10B	0.9600
C1—C2	1.337 (4)	C10—H10C	0.9600
C1—C11	1.478 (4)	C11—C12	1.512 (4)
C2—C3	1.458 (4)	C11—H11A	0.9700
C2—H2	0.9300	C11—H11B	0.9700
C3—C4	1.325 (4)	C12—C13	1.492 (5)
C3—H3	0.9300	C12—H12A	0.9700
C4—C5	1.475 (4)	C12—H12B	0.9700
C5—C6	1.519 (4)	C13—C14	1.539 (5)
C5—H5A	0.9700	C13—H13A	0.9700
C5—H5B	0.9700	C13—H13B	0.9700
C6—C7	1.488 (5)	C14—C15	1.443 (6)
C6—H6A	0.9700	C14—H14A	0.9700
C6—H6B	0.9700	C14—H14B	0.9700
C7—C8	1.529 (5)	C15—C16	1.533 (7)
C7—H7A	0.9700	C15—H15A	0.9700
C7—H7B	0.9700	C15—H15B	0.9700
C8—C9	1.446 (7)	C16—H16A	0.9600
C8—H8A	0.9700	C16—H16B	0.9600
C8—H8B	0.9700	C16—H16C	0.9600
C9—C10	1.487 (7)		
			
O1—S1—O2	116.66 (12)	C8—C9—H9B	108.4
O1—S1—C1	110.71 (13)	C10—C9—H9B	108.4
O2—S1—C1	110.61 (13)	H9A—C9—H9B	107.5
O1—S1—C4	111.64 (13)	C9—C10—H10A	109.5
O2—S1—C4	110.35 (13)	C9—C10—H10B	109.5
C1—S1—C4	94.74 (13)	H10A—C10—H10B	109.5
C2—C1—C11	133.4 (3)	C9—C10—H10C	109.5
C2—C1—S1	106.8 (2)	H10A—C10—H10C	109.5
C11—C1—S1	119.78 (19)	H10B—C10—H10C	109.5
C1—C2—C3	115.5 (2)	C1—C11—C12	113.9 (2)
C1—C2—H2	122.3	C1—C11—H11A	108.8
C3—C2—H2	122.3	C12—C11—H11A	108.8
C4—C3—C2	115.9 (2)	C1—C11—H11B	108.8
C4—C3—H3	122.0	C12—C11—H11B	108.8
C2—C3—H3	122.0	H11A—C11—H11B	107.7
C3—C4—C5	133.2 (3)	C13—C12—C11	114.4 (3)
C3—C4—S1	107.0 (2)	C13—C12—H12A	108.6
C5—C4—S1	119.80 (19)	C11—C12—H12A	108.6
C4—C5—C6	113.8 (3)	C13—C12—H12B	108.6
C4—C5—H5A	108.8	C11—C12—H12B	108.6
C6—C5—H5A	108.8	H12A—C12—H12B	107.6
C4—C5—H5B	108.8	C12—C13—C14	112.9 (3)
C6—C5—H5B	108.8	C12—C13—H13A	109.0
H5A—C5—H5B	107.7	C14—C13—H13A	109.0
C7—C6—C5	114.3 (3)	C12—C13—H13B	109.0
C7—C6—H6A	108.7	C14—C13—H13B	109.0
C5—C6—H6A	108.7	H13A—C13—H13B	107.8
C7—C6—H6B	108.7	C15—C14—C13	115.7 (4)
C5—C6—H6B	108.7	C15—C14—H14A	108.4
H6A—C6—H6B	107.6	C13—C14—H14A	108.4
C6—C7—C8	113.6 (3)	C15—C14—H14B	108.4
C6—C7—H7A	108.8	C13—C14—H14B	108.4
C8—C7—H7A	108.8	H14A—C14—H14B	107.4
C6—C7—H7B	108.8	C14—C15—C16	111.9 (5)
C8—C7—H7B	108.8	C14—C15—H15A	109.2
H7A—C7—H7B	107.7	C16—C15—H15A	109.2
C9—C8—C7	114.6 (4)	C14—C15—H15B	109.2
C9—C8—H8A	108.6	C16—C15—H15B	109.2
C7—C8—H8A	108.6	H15A—C15—H15B	107.9
C9—C8—H8B	108.6	C15—C16—H16A	109.5
C7—C8—H8B	108.6	C15—C16—H16B	109.5
H8A—C8—H8B	107.6	H16A—C16—H16B	109.5
C8—C9—C10	115.5 (5)	C15—C16—H16C	109.5
C8—C9—H9A	108.4	H16A—C16—H16C	109.5
C10—C9—H9A	108.4	H16B—C16—H16C	109.5

### Hydrogen-bond geometry (Å, º)

**Table:**

*D*—H···*A*	*D*—H	H···*A*	*D*···*A*	*D*—H···*A*
C3—H3···O2^i^	0.93	2.54	3.186 (3)	126

Symmetry code: (i) −*x*+3/2, *y*−1/2, −*z*+1/2.
